# Prevalence of mental health problems and associated factors among front-line public health workers during the COVID-19 pandemic in China: an effort–reward imbalance model-informed study

**DOI:** 10.1186/s40359-021-00563-0

**Published:** 2021-04-12

**Authors:** Jing Zhang, Yijing Wang, Jingdong Xu, Hua You, Yan Li, Yuan Liang, Shan Li, Lina Ma, Joseph Tak-fai Lau, Yuantao Hao, Shilin Chen, Jing Zeng, Jinghua Li, Jing Gu

**Affiliations:** 1 School of Public Health, Sun Yat-Sen University, Guangzhou, 510080 China; 2 Sun Yat-Sen University Global Health Institute, School of Public Health and Institute of State Governance, Sun Yat-Sen University, Guangzhou, 510080 China; 3 Hubei Province Center for Disease Control and Prevention, Wuhan, 430097 China; 4 School of Public Health, Nanjing Medical University, Nanjing, 210000 China; 5 Guangzhou Center for Disease Control and Prevention, Guangzhou, 510440 China; 6 School of Public Health, Huazhong University of Science and Technology, Wuhan, 430074 China; 7 Zigong Center for Disease Control and Prevention, Zigong, 643000 China; 8 Centre for Health Behaviors Research, JC School of Public Health and Primary Care, The Chinese University of Hong Kong, Hong Kong, China

**Keywords:** Effort–reward imbalance, Reward, Depression, Anxiety, Public health worker

## Abstract

**Background:**

Poor mental health status and associated risk factors of public health workers have been overlooked during the COVID-19 pandemic. This study used the effort–reward imbalance model to investigate the association between work-stress characteristics (effort, over-commitment, reward) and mental health problems (anxiety and depression) among front-line public health workers during the COVID-19 pandemic in China.

**Methods:**

A total of 4850 valid online questionnaires were collected through a self- constructed sociodemographic questionnaire, the adapted ERI questionnaire, the 9-item Patient Health Questionnaire (PHQ-9) and the 7-item General Anxiety Disorder Scale (GAD-7). Hierarchical logistic regression analysis was conducted to investigate the association between ERI factors and mental health problems (i.e., depression and anxiety), with reward treated as a potential moderator in such associations.

**Results:**

The data showed that effort and over-commitment were positively associated with depression and anxiety, while reward was negatively associated with depression and anxiety. Development and job acceptance were the two dimensions of reward buffered the harmful effect of effort/over-commitment on depression and anxiety, whereas esteem was non-significant.

**Conclusions:**

This study confirmed the harmful effects of effort and over-commitment on mental health among public health workers during the COVID-19 pandemic in China. Such effects could be alleviated through an appropriate reward system, especially the development and job acceptance dimensions of such a system. These findings highlight the importance of establishing an emergency reward system, comprising reasonable work-allocation mechanism, bonuses and honorary titles, a continuous education system and better career-development opportunities.

**Supplementary Information:**

The online version contains supplementary material available at 10.1186/s40359-021-00563-0.

## Background

On March 11, 2020, due to the worldwide spread of SARS-CoV-2, the virus that can lead to COVID-19, and the associated morbidity and mortality from this disease, the World Health Organization declared COVID-19 a pandemic [1]. As of the time of writing, 8 May 2020, a total of 84,395 and 3,767,744 confirmed cases had been reported in China [2] and worldwide [3] and at this point, the COVID-19 pandemic is under control in China, with only 208 confirmed cases existing on 8 May [2]. This could not have been achieved without the immense effort and commitment from medical workers and public health workers. Chinese public health workers, who work at the Chinese Centre for Disease Control and Prevention (China CDC) or primary health care institutes (PHIs), made various contributions to prevent and control the spread of COVID-19, such as epidemiological investigation, surveillance, professional technical guidance, specimen collection and examination, health education and report epidemic data [4, 5]. These workers are therefore an indispensable section of the anti-pandemic effort.

During public health emergencies, such as those resulting from the SARS and MERS epidemics and the COVID-19 pandemic, public health workers and medical workers face numerous stressors such as risk of infection, heavy workload, inadequate equipment and social support [4, 6], and are consequently at high risk of developing mental health problems. The mental health and associated risk factors of medical workers have typically been well studied and documented during epidemics such as SARS[7, 8] and MERS [9, 10], and the ongoing COVID-19 pandemic[11, 12]. However, the mental health status and associated risk factors of public health workers have been overlooked: we identified only one study [4] that has examined the prevalence of mental health problems among public health workers during the COVID-19 pandemic. This study found that the prevalence of probable depression and anxiety among public health workers during the pandemic in China was 21.3% and 19.0%, respectively. Poor mental health status is known to be significantly associated with low work efficiency, excess lost productive time, poor physical health and poor quality of life [13–15], all of which may detract from the effectiveness of workers’ COVID-19 prevention and control work.

To design effective interventions to reduce mental health problems among public health workers, factors associated with mental health problems must be investigated. In the context of public health emergencies, working conditions and stress-related factors are among the most important determinants of mental health status among public health workers facing demanding work. The effort–reward imbalance model (ERI) is commonly used in research on work stress and working conditions in many professional populations, such as nurses [16], teachers [17, 18], police officers [19] and doctors [20]. Additionally, the ERI model has been widely used to predict many stress-related physical and psychological disorders, such as hypertension [21, 22], depression [16, 23] and anxiety [16].

The ERI model includes two extrinsic components: extrinsic effort in work and extrinsic rewards of money, esteem, job promotion and job security [24]. The model also includes an intrinsic component, namely over-commitment, a motivational pattern of excessively high job involvement [25]. ERI theory posits that failed reciprocity, such as high extrinsic effort and over-commitment by workers leading to low reward, likely generates strain and negative health outcomes in workers [24, 26]. Previous studies found that occupational groups, such as public health workers who had excessive work-related motivations and attitudes without alternative, non-work-related foci, will frequently suffer from failed reciprocity [27], leading to a higher risk of developing mental health problems[4]. Thus, the ERI model is an appropriate tool to use for investigating the association between work stress factors (i.e., effort, reward and over-commitment) and mental health problems (i.e., depression and anxiety) in front-line public health workers in the COVID-19 pandemic, as these workers have been greatly overburdened and under extreme pressure.

In the traditional ERI model, over-commitment is often used as a moderator for the association between ERI and health outcomes, with the assumption that over-commitment could strengthen the negative effect of ERI on health outcomes [28, 29]. Under the special circumstance of the COVID-19 pandemic, China has rapidly and fully mobilized its public health workforce. Many municipalities began to call their public health workers into the pandemic control program before cases were reported in their areas; later, during the Chinese New Year, the participation rate increased substantially to 90% [4]. During public health emergencies, it is inevitable that public health workers must work extremely hard and are subject to over-commitment, while rewards for these efforts are relatively flexible, and can be adjusted to optimally compensate workers. Therefore, in this study we explored the moderating effect of reward.

We used the ERI model to investigate the association between work stress factors (i.e., effort, reward and over-commitment) and mental health problems (i.e., depression and anxiety) in front-line public health workers during the COVID-19 pandemic in China. We hypothesized that (1) effort and over-commitment would be risk factors for depression and anxiety among public health workers, (2) reward would be a protective factor for depression and anxiety among public health workers, and (3) reward would reduce the harmful effect of effort and over-commitment on depression and anxiety.

## Methods

### Study design and population

From 18 February 2020 to 1 March 2020, this cross-sectional study was conducted in five provinces (Hubei, Guangdong, Sichuan, Jiangsu and Gansu), representing different regions of China and different severities of the COVID-19 epidemic. Moreover, 3–5 cities in each province, 3–5 districts/counties in each city and 5–10 sub-districts/towns in each district were further selected by a similar procedure to achieve a representative sample of different local regions and areas experiencing different severities of the pandemic. In this study, online questionnaires (shown in Additional file 1: Table S1) were collected and distributed by site collaborators at each selected center via working groups on WeChat and QQ (the most commonly used social networking applications in China).

The eligible participants’ criteria are as follows, (1) aged 18 years or above, (2) working at a Center for Disease Control (CDC) (at province, city and district/county levels) or public health institute (PHI) (at sub-district/town level) of the selected places during the survey period, and (3) had participated in COVID-19-related work. All eligible participants were clearly briefed about the background, aims, anonymous nature and length (about 8–12 min to complete) of the survey before self-administering the questionnaire. Their informed consents were then obtained online by clinking the questionnaire link.

### Measurement

#### Socio-demographic characteristics

Information was collected on workers’ age (by year), sex (male, female), job title (junior, intermediate, vice-senior/senior and other (e.g., volunteers)) and whether they had children under 6 years of age.

#### Work stress

In this study, occupational stressors were evaluated by the ERI model [24]. The Chinese version of the ERI has been used to assess various professional populations [22, 30], and has shown good internal reliability. The items of the ERI questionnaire were adjusted to fit the characteristics of the pandemic background. The adjustments made are shown in Table 1.

**Table 1:** The adjustment of the effort–reward imbalance questionnaire items

Subscale	Original questionnaire		Adapted questionnaire	
	Item	Content	Item	Content
Effort				
	ERI-1	I have constant time-pressure due to a heavy workload	E-1	I have constant time-pressure due to a heavy workload
	ERI-2	I have many interruptions and disturbances in my job	E-2	I sacrifice a lot for pandemic-related work
	ERI-3	I have a lot of responsibility in my job	E-3	I have a lot of responsibility in pandemic-related work
	ERI-4	I am often pressured to work overtime	E-4	I need to work overtime for pandemic-related work
	ERI-5	My job is physically demanding		
	ERI-6	Over the past few years, my job has become more and more demanding		
Reward				
	Job promotion		Development	
	ERI-11	My job-promotion prospects are poor	R-1	Participating in pandemic-related work will improve my ability
	ERI-14	My current occupational position adequately reflects my education and training	R-2	Participating in pandemic-related work will help my future development
	ERI-16	Considering all of my efforts and achievements, my work prospects are adequate		
	ERI-17	Considering all of my efforts and achievements, my salary/income is adequate		
	Esteem		Esteem	
	ERI-7	I receive the respect I deserve from my superiors	R-3	I receive the respect I deserve from my superiors
	ERI-8	I receive the respect I deserve from my colleagues	R-4	I receive the respect I deserve from my colleagues
	ERI-9	I experience adequate support in difficult situations	R-5	I receive the respect I deserve from my service objects
	ERI-10	I am treated unfairly at work	R-6	I receive the respect I deserve from society
	ERI-15	Considering all of my efforts and achievements, I receive the respect and prestige I deserve		
	Job security		Job acceptance	
	ERI-12	I have experienced or I expect to experience an undesirable change in my work situation	R-7	I participate in noble work
	ERI-13	My job security is poor	R-8	The work I am engaged in has important social significance
			R-9	To be required to participate in pandemic-related work reflects your ability
Over-commitment				
	OC-1	I am easily overwhelmed by time-pressures at work	OC-1	I am often faced with unsolvable difficulties in pandemic-related work
	OC-2	As soon as I get up in the morning, I start thinking about work problems	OC-2	As soon as I get up in the morning, I start thinking about work problems
	OC-3	When I get home, I can easily relax and “switch off” work	OC-3	When I get home, I can easily relax and “switch off” pandemic-related work
	OC-4	People close to me say I sacrifice too much for my job	OC-4	People close to me say I sacrifice too much for my job
	OC-5	Work rarely lets me go; it is still on my mind when I go to bed	OC-5	Pandemic-related work rarely lets me go; it is still on my mind when go to bed
	OC-6	If I postpone something that I was supposed to do today, I’ll have trouble sleeping at night		

All of the items were measured on a Likert-type response scale ranging from 1 (“strongly disagree”) to 5 (“strongly agree”). The effort scale contained four items and the total score ranged from 5 to 20, where a higher score reflected that more effort was made. The 9-item reward scale was composed of three dimensions: esteem (four items), development (two items) and job acceptance (three items). The total score ranged from 5 to 45, with a higher score reflecting greater reward. The over-commitment scale contained five items, with the total score ranging from 5 to 25.

In this study, the Cronbach’s alpha value (internal reliability) for the adapted ERI scale was 0.855 (higher than for the original ERI, 0.74–0.81). The exploratory factor analysis (EFA) identified five components (effort, development, esteem, job acceptance, over-commitment) with eigenvalues of greater than 1, explaining 15.65%, 10.12%, 15.90%, 13.05% and 16.15% of the variance, respectively, and accounting for 70.87% of the total variance (shown in Additional file 1: Table S2). The confirmatory factor analysis (CFA) identified that the factorial structure of the adapted questionnaire had satisfactory convergent validity and discriminant validity and a good model fit (RMSEA = 0.071, CFI = 0.930, IFI = 0.930, TLI = 0.915) (shown in Additional file 1: Table S3–S5).

#### Depression

In this study, the presence of depression was evaluated by the Chinese version of the 9-item patient health questionnaire (PHQ-9). The PHQ-9 scale has been widely used in various Chinese populations including medical staff, with good reliability (Cronbach α: 0.86–0.873) and internal validity (0.854–0.86) [31–34].Participants were asked their depressive symptoms in the past two weeks, on a 4-point Likert scale for each item, ranging from 0 (“never”) to 3 (“nearly every day”). The total score ranged from 0 to 27 points, with a higher score reflecting greater depression severity. A score of 10 or more was classified as a major depressive disorder, with a sensitivity of 80% and specificity of 92% [35, 36]. The Cronbach’s alpha value was 0.92 in this study.

#### Anxiety

In this study, the presence of anxiety was evaluated by the 7-item General Anxiety Disorder scale (GAD-7) [37]. The GAD-7 scale has been widely used in various Chinese populations including medical staff, with good reliability (Cronbach α: 0.91) and internal validity (0.76–0.86) [31, 32, 38]. Participants were asked their anxious symptoms in the past two weeks, on a 4-point Likert scale for each item, ranging from 0 (“never”) to 3 (“often”). The total score ranged from 0 to 21 points, with a higher score reflecting greater anxiety severity. The cutoff point of 10 or above was used to define a probable case of moderate anxiety disorder, with a sensitivity of 89% and specificity of 82% [37, 39, 40]. The Cronbach’s alpha value was 0.94 in this study.

### Statistical analysis

The continuous variables (age, effort, reward and over-commitment), which obeyed a normal distribution, were described using means (and standard deviations). The categorical variables (sex, job title, having children under 6 years, depression and anxiety) were described using frequencies (percentages). Hierarchical logistic regression analysis was conducted to investigate the association between ERI factors and mental health problems (depression and anxiety), with reward as a potential moderator in such associations.

First, we investigated the relationship between sociodemographic factors and mental health problems using Logistic regression (Model 1). Second, we examined the main effects of ERI factors on mental health, after controlling for sociodemographic factors (Model 2). Third, to examine whether the relationship between effort and mental health problems was moderated by reward, an interaction term was added between effort and reward in Model 2, to form Model 3. Fourth, an interaction term was similarly added between over-commitment and reward in Model 2, to test the buffering effect of reward on the association between over-commitment and mental health problems, to form Model 4. Further analyses were carried out with each of the three dimensions of reward as independent variables, repeating the analysis of Models 2–4. The correlation coefficient Odds ratio (OR) and 95% confidence intervals (CIs) were reported. Stata MP 14.0 (College Station, TX, USA, 2014) was used for data analysis. Significance referred to *P* values < 0.05.

## Results

Of the 7090 completed questionnaires, 528 (7.4%) did not pass the consistency checks (i.e., the reported number of working overtime days was greater than the number of working overnight days), 245 (3.4%) did not report any COVID-19-related work and 1467 (20.7%) from Guangdong province contained uncompleted depression and anxiety sections, as these were set as optional sections for participants in Guangdong due to the length of the questionnaire. Finally, a total of 4850 (68.4%) participants were included in the analysis (shown in Table 2). The 2019 China Health Statistics Yearbook [41] showed that 71.8% of Chinese health workers were female and 28.2% were male, and 65.3% were aged 25–44 years. Regarding the distribution of job titles, 62.1% were junior, 20.1% were intermediate and 8% were senior. According to those statistics, the distribution of key socio-demographic characteristics in our sample is similar to that among health workers nationwide.

**Table 2:** Background characteristics of the participants (N = 4850)

Variable	N (%)/Mean (SD)
**Socio-demographic characteristics**	
Sex	
Male	1800 (37.11%)
Female	3050 (62.89%)
Age	38.86 (9.74)
Having children under 6 years	
No	3608 (74.39%)
Yes	1242 (25.61%)
Job title	
Junior	2329 (48.02%)
Intermediate	1381 (28.47%)
Senior	506 (10.43%)
Other (e.g., volunteers)	634 (13.07%)
**Effort–reward**	
Effort (score range: 4–20)	12.99 (2.80)
Over-commitment (score range: 5–25)	14.06 (2.57)
Reward (score range: 9–45)	33.44 (4.65)
Esteem (score range:4–20)	13.06 (2.64)
Development (score range: 2–10)	7.66 (1.52)
Job acceptance (score range: 3–15)	12.72 (1.91)
**Mental health problems**	
PHQ-9 (score range: 0–27)	5.94 (5.59)
Depression	
No	3816 (78.68%)
Yes	1034 (21.32%)
GAD-7 (score range: 0–21)	5.69 (5.07)
Anxiety	
No	3930 (78.68%)
Yes	920 (18.97%)

Table 3 shows the results of hierarchical logistic regressions for depression. First, Model 1 assessed the relationship between socio-demographic variables and depression. Significant variables were age (OR: 0.97; 95% CI: 0.96, 0.98) and having a senior job title (OR: 1.35; 95% CI: 1.04, 1.76). After adjustment for all socio-demographic variables, Model 2 showed that effort (OR: 1.33; 95% CI: 1.29, 1.38) and over-commitment (OR: 1.19; 95% CI: 1.15, 1.23) had a positive association with depression, whereas reward (OR: 0.91; 95% CI: 0.89, 0.92) had a negative association with depression. However, neither the interaction between reward and effort in Model 3 nor the interaction between reward and over-commitment in Model 4 was significant for depression.

**Table 3:** Logistic regression analysis of effort/over-commitment and reward on depression (N = 4850)

Depression	Model 1		Model 2		Model 3		Model 4	
	*OR*	95%*Cl*	*OR*	95%*Cl*	*OR*	95%*Cl*	*OR*	95%*Cl*
**Socio-demographic characteristics**								
Sex								
Male	1.00	–	1.00	–	1.00	–	1.00	–
Female	1.09	(0.94,1.27)	1.47	(1.24,1.73) ***	1.47	(1.24,1.74) ***	1.46	(1.24,1.73) ***
Age	0.97	(0.96,0.98)***	0.96	(0.95, 0.97)***	0.96	(0.95, 0.97)***	0.96	(0.95,0.97)***
Having children under 6 years of age								
No	1.00	–	1.00	–	1.00	–	1.00	–
Yes	1.17	(0.99, 1.37)	1.02	(0.85,1.22)	1.02	(0.86,1.22)	1.02	(0.86,1.39)
Job title								
Junior	1.00	–	1.00	–	1.00	–	1.00	–
Intermediate	1.32	(1.11, 1.57)	1.16	(0.96, 1.39)	1.15	(0.96, 1.39)	1.15	(0.95, 1.39)
Senior	1.35	(1.04, 1.76)*	1.18	(0.88, 1.57)	1.18	(0.89, 1.57)	1.18	(0.89, 1.57)
Others	1.14	(0.92, 1.42)	1.19	(0.94, 1.50)	1.19	(0.94, 1.50)	1.19	(0.94, 1.50)
**Effort–reward**								
Effort			1.33	(1.29, 1.38)***	1.43	(1.18, 1.73)***	1.33	(1.29, 1.38)***
Over–commitment			1.19	(1.15, 1.23)***	1.19	(1.15, 1.23)***	1.34	(1.09, 0.06)***
Reward			0.91	(0.89, 0.92)***	0.94	(0.86, 1.02)	0.96	(0.87, 1.05)
**Interaction item**								
Effort × reward					1.00	(0.99, 1.00)		
Over-commitment × reward							1.00	(0.99,1.00)
F-statistics		79.40***		782.11***		782.66***		783.44***
Adjusted R^2^		1.58%		15.56%		15.57%		15.59%

^*^ *P* < 0.05; ***P < 0.001

Table 4 shows the results of hierarchical logistic regressions for anxiety. First, Model 1 assessed the relationship between socio-demographic variables and anxiety. The one significant variable was having children under 6 years of age (OR: 1.30; 95% CI: 1.10, 1.54). After adjustment for all socio-demographic variables, Model 2 showed that effort (OR: 1.33; 95% CI: 1.31, 1.41) and over-commitment (OR: 1.19; 95% CI: 1.30, 1.40) had a positive association with anxiety, whereas reward (OR: 0.91; 95% CI: 0.91, 0.94) had a negative association with anxiety. Model 3 investigated the interaction between effort and reward in explaining the variance in anxiety among public health workers during the pandemic. A weak, significant and negative interaction (OR: 0.99; 95% CI: 0.99, 1.00) was found. Similarly, Model 4 found that reward had a weak significant moderating effect (OR: 0.99; 95% CI: 0.98, 1.00) on the association between over-commitment and anxiety.

**Table 4:** Logistic regression analysis of effort/over-commitment and reward on anxiety (N = 4850)

Anxiety	Model 1		Model 2		Model 3		Model 4	
	*OR*	95%*Cl*	*OR*	95%*Cl*	*OR*	95%*Cl*	*OR*	95%*Cl*
**Socio-demographic characteristics**								
Sex								
Male	1.00	–	1.00	–	1.00	–	1.00	–
Female	1.05	(0.90,1.23)	1.56	(1.30,1.87) ***	1.57	(1.31,1.88) ***	1.56	(1.30,1.87) ***
Age	0.99	(0.98,1.00)	0.98	(0.97, 0.99)***	0.98	(0.97, 0.99)***	0.98	(0.97,0.99)***
Having children under 6 years of age								
No	1.00	–	1.00	–	1.00	–	1.00	–
Yes	1.30	(1.10, 1.54)***	1.12	(0.93,1.36)	1.13	(0.93,1.37)	1.14	(0.71,1.06)
Job title								
Junior	1.00	–	1.00	–	1.00	–	1.00	–
Intermediate	1.06	(0.89, 1.26)	0.87	(0.71, 1.06)	0.87	(0.71, 1.06)	0.97	(0.71, 1.06)
Senior	0.87	(0.66, 1.15)	0.67	(0.49, 0.91)	0.67	(0.49, 0.92)^*^	0.67	(0.49, 0.92)^*^
Others	1.02	(0.82, 1.28)	1.05	(0.82, 1.35)	1.05	(0.82, 1.35)	1.05	(0.82, 1.35)
**Effort–reward**								
Effort			1.33	(1.31, 1.41)***	1.73	(1.39, 2.17)***	1.33	(1.31, 1.41)***
Over-commitment			1.19	(1.30, 1.40)***	1.35	(1.30, 1.40)***	1.34	(1.39, 2.33)***
Reward			0.91	(0.91, 0.94)***	1.03	(0.93, 1.13)	0.96	(0.94, 1.17)
**Interaction item**								
Effort × reward					0.99	(0.99, 1.00)*		
Over-commitment × reward							0.99	(0.98,1.00)*
F-statistics		22.18***		975.54***		980.32***		980.65***
Adjusted R^2^		0.47%		20.70%		20.80%		20.81%

^*^P < 0.05; ***P < 0.001

Further analyses were conducted with each of the three dimensions of reward as independent variables (shown in Additional file 1: Table S6–S11). The results showed that the significance of the moderating effects varied by reward dimension. For both depression and anxiety, the moderating effect of reward was mainly reflected in the development and job acceptance dimensions, whilst the moderating effect of esteem was non-significant in the association between effort/over-commitment and depression/anxiety.

## Discussion

In this study an ERI model was used to measure the associations of work stress (effort, reward and over-commitment) with mental health problems (depression and anxiety) among 4850 Chinese front-line public health workers involved in healthcare response to the COVID-19 pandemic. Notably, effort and over-commitment were positively associated with depression and anxiety (Hypothesis 1), while reward was negatively associated with depression and anxiety (Hypothesis 2). Additionally, it was also found that reward, especially for the development and job acceptance dimensions, could alleviate the harmful effect of effort and over-commitment on both depression and anxiety, whereas esteem was non-significant. (Hypothesis 3).

The data showed that effort and over-commitment were risk factors for depression and anxiety among public health workers. This was in line with findings from previous studies [16, 42, 43], where over-commitment and extrinsic effort were significantly positively associated with anxiety and depression disorders. Front-line public health workers were inevitably required to exert immense effort and were often overcommitted at work during this time, due to the lack of a professional workforce, and the heavy workload and pressure from the public to curb the pandemic. Such circumstances may evoke common psychological phenomena of entrapment and learned helplessness, which may lead to mental disorders [44–46]. Consequently, more resources should be allocated to public health systems and more attention should be paid to the training of public health workers in China, to ensure that there is a sufficiently large, high-quality and effective public health workforce. With a better-trained workforce, it will be possible to partly alleviate workers’ risk of exposure to intensive workload and pressure, thereby improving their mental health status and work effectiveness during public health emergencies.

Under the special circumstance of the COVID-19 pandemic, we used an adjusted ERI model with reward as a moderating variable. The results showed that reward was beneficial for mental health through both direct and indirect mechanisms, as follows: (1) participants with higher reward scores had a lower risk of depression and anxiety disorder; and (2) through further analysis, development and job acceptance were the two dimensions of reward that buffered the harmful effect of effort/over-commitment on depression and anxiety. However, it is worth noting that the adjusted R squared differences after the addition of the interaction terms were tiny, and the odds ratios (OR) and confidence intervals of the interaction terms were close to 1. Thus, we cannot rule out that the significance may have been due to confounding effects, and we caution against over-interpreting the moderating effect.

By participating in COVID-19 prevention and control work, public health workers in China have already been partly exposed to some dimensions of reward. For instance, they were provided with a range of training sessions to learn specific technical skills to cope with an emergency like COVID-19 [4], which is an important part of the development dimension of reward, in that skills acquisition and career development may enhance employees’ mental health [47]. Job acceptance is reflected in employees’ display of personal expertise at work and in the meaning of the work itself. Specifically, enabling employees to use their strengths can help them to cultivate a positive mindset and further improve their resilience to emergencies; this is a widely used technique in strength-based therapy [48], and meaningful work has been linked to lower depression and anxiety [49]. Although no moderating effect of the esteem dimension was found, previous research showed that esteem—as reflected by respect and considered as a social need—was nevertheless a protective factor for mental health [50]. Thus, esteem should also be addressed in a reward system.

Our findings imply that when immense effort and over-commitment are required of public health workers, improving their rewards system will be crucial for alleviating any mental health problems they may develop. This reward system could be used as a reference system for use in other COVID-19 emergencies (especially as COVID-19 may become a long-standing disease that coexists with humans [51]) or adapted for use in other public health emergencies.

In China, the career development of grassroots public health workers is difficult because of a lack of continuing education. Moreover, the social status, income and professional sense of honor of public health workers are at a low level [52]. Based on the current difficulties, the following suggestions on developing a reward system are proposed.

One of the important challenges faced by policymakers when developing a multi-level short-term reward system is how to ensure its fairness, objectivity and transparency. An internal approach could involve the related department establishing an optimized work-allocation mechanism that enables public health workers to use their strengths in their work. An external approach could be to provide bonuses, awards or subsidies, or to confer honorary titles to enhance workers’ professional sense of honor. These are relative commonly used and effective reward measures, which have been adopted to varying degrees by China and some foreign countries during COVID-19 [53–55].

For a long-term reward system, a key internal approach is to establish a continuing education system to continuously improve individual employees’ professional abilities. Externally, the function and importance of public health should be publicized, to increase societal recognition of public health and provide better career-development opportunities for experienced and capable public health workers [52, 56].

This timely cross-sectional study has several strengths. First, it included the sample of Chinese front-line public health workers in the COVID-19 pandemic, who were recruited from different regions of China with different levels of pandemic severity. Additionally, this theory-based study is one of the first to focus on the mental health problems and associated factors among public health workers during the COVID-19 pandemic.

However, several limitations warrant mention. First, because non-individualised URLs and convenience sampling were applied in our study, we do not know the total numbers of CDC workers or PHI workers that we could have reached. Thus, information about non-participation is not available and the response rate of the targeted centres may not have been high. Second, the measurements of the ERI questionnaire were tailored to the characteristics of the pandemic background, and were not validated in previous studies. Although we supplemented the adapted version of the ERI questionnaire with a confirmatory factor analysis, which reflected a good convergent validity, discriminant validity and model fit. However, the reward items of the questionnaire did not include financial rewards (e.g., wages and bonuses), which are more difficult to obtain. Therefore, it may have underestimated the strength of the association between reward and mental health problems. Moreover, some confounding factors on mental health (e.g., history of mental disorders) were not investigated in our study due to limited length for the questionnaire. Additionally, due to time constraints, the protocol and data analysis plan were not preregistered before the investigation. To reduce the risk of subjective bias in model estimation, and to avoid the possibility of data-trawling or p-hacking, we used a hierarchical regression analysis in which variables were entered into the model in a pre-determined order based on the underlying effort-reward imbalance theory. Nonetheless, some caution should be exercised in generalizing our findings given the lack of pre-registration.

## Conclusions

In summary, our results confirmed the harmful effects of immense effort and over-commitment on the mental health of public health workers during the COVID-19 pandemic in China. Reward, especially its development and job-acceptance dimensions, was found to be a protective factor and could alleviate the negative effect of effort and over-commitment on mental health. Our findings show that the system used to train public health workers in China should be improved, as this would enhance public health by ensuring the generation of a sufficiently large, high-quality and effective public health workforce. It is also essential to establish short-term and long-term reward systems that incorporate comprehensive reward dimensions, such as development, job acceptance and esteem.

## Supplementary Information

**Additional file 1**. The English version of online survey questionnaires, the confirmatory factor analysis and the exploratory factor analysis of ERI, and the supplementary analysis of results.

## Data Availability

The data that support the findings of this study are available from the corresponding author upon reasonable request.

## Abbreviations

CI: Confidence interval
COVID-19: Coronavirus disease 2019
ERI: Effort–reward imbalance

## References

[1] World Health Organization: WHO Director-General's opening remarks at the media briefing on COVID-19–11 March 2020. In*.* World Health Organization; 2020.

[2] National Health Commission of the People’s Republic of China: Updates on COVID-19 epidemic In. National Health Commission of the People’s Republic of China; 2020.

[3] World Health Organization: Novel coronavirus(NCOVID-19) situation. In.World Health Organization; 2020.

[4] Li J, Xu J, Zhou H, You H, Wang X, Li Y. Working condition and health status of 6317 front line public health workers during the COVID-19 epidemic across 5 provinces in China: a cross-sectional study. Bull World Health Organ 2020. 10.1186/s12889-020-10146-0 PMC7794632 33422035

[5] National Health Commission of the People’s Republic of China: Protocol for Prevention and Control of COVID-19 (Edition 6) (in Chinese). In*.*National Health Commission of the People’s Republic of China; 2020. 10.46234/ccdcw2020.082 PMC8392946 34594648

[6] Brooks SK, Dunn R, Amlôt R, Rubin GJ, Greenberg N (2018). A Systematic, thematic review of social and occupational factors associated with psychological outcomes in healthcare employees during an infectious disease outbreak. J Occup Environ Med.

[7] Chong M-Y, Wang W-C, Hsieh W-C, Lee C-Y, Chiu N-M, Yeh W-C, Huang T-L, Wen J-K, Chen C-L (2004). Psychological impact of severe acute respiratory syndrome on health workers in a tertiary hospital. Br J Psychiatry.

[8] Wu P, Fang Y, Guan Z, Fan B, Kong J, Yao Z, Liu X, Fuller CJ, Susser E, Lu J (2009). The psychological impact of the SARS epidemic on hospital employees in China: exposure, risk perception, and altruistic acceptance of risk. Can J Psychiatry.

[9] Khalid I, Khalid TJ, Qabajah MR, Barnard AG, Qushmaq IA (2016). Healthcare workers emotions, perceived stressors and coping strategies during a MERS-CoV outbreak. Clin Med Res.

[10] Lee SM, Kang WS, Cho AR, Kim T, Park JK (2018). Psychological impact of the 2015 MERS outbreak on hospital workers and quarantined hemodialysis patients. Compr Psychiatry.

[11] Greenberg N, Docherty M, Gnanapragasam S, Wessely S (2020). Managing mental health challenges faced by healthcare workers during covid-19 pandemic. BMJ.

[12] Xiang YT, Jin Y, Wang Y, Zhang Q, Zhang L, Cheung T (2020). Tribute to health workers in China: a group of respectable population during the outbreak of the COVID-19. Int J Biol Sci.

[13] Asante JO, Li MJ, Liao J, Huang YX, Hao YT (2019). The relationship between psychosocial risk factors, burnout and quality of life among primary healthcare workers in rural Guangdong province: a cross-sectional study. BMC Health Serv Res.

[14] Jain G, Roy A, Harikrishnan V, Yu S, Dabbous O, Lawrence C (2013). Patient-reported depression severity measured by the PHQ-9 and impact on work productivity: results from a survey of full-time employees in the United States. J Occup Environ Med.

[15] Stewart WF, Ricci JA, Chee E, Hahn SR, Morganstein D (2003). Cost of lost productive work time among US workers with depression. JAMA.

[16] Mark G, Smith AP (2012). Occupational stress, job characteristics, coping, and the mental health of nurses. Br J Health Psychol.

[17] Bellingrath S, Rohleder N, Kudielka BM (2013). Effort-reward-imbalance in healthy teachers is associated with higher LPS-stimulated production and lower glucocorticoid sensitivity of interleukin-6 in vitro. Biol Psychol.

[18] Ren C, Li X, Yao X, Pi Z, Qi S (2019). Psychometric properties of the effort-reward imbalance questionnaire for teachers (teacher ERIQ). Front Psychol.

[19] Violanti JM, Fekedulegn D, Gu JK, Allison P, Mnatsakanova A, Tinney-Zara C, Andrew ME (2018). Effort-reward imbalance in police work: associations with the cortisol awakening response. Int Arch Occup Environ Health.

[20] von dem Knesebeck O, Klein J, Grosse Frie K, Blum K, Siegrist J (2010). Psychosocial stress among hospital doctors in surgical fields: results of a nationwide survey in Germany. Dtsch Arztebl Int.

[21] Peter R, Siegrist J (1997). Chronic work stress, sickness absence, and hypertension in middle managers: general or specific sociological explanations?. Soc Sci Med.

[22] Xu W, Yu H, Hang J, Gao W, Zhao Y, Guo L (2013). The interaction effect of effort-reward imbalance and overcommitment on hypertension among Chinese workers: findings from SHISO study. Am J Ind Med.

[23] de Araújo TM, Siegrist J, Moreno AB, de Jesus Mendes da Fonseca M, Barreto SM, Chor D, Griep RH. Effort-reward imbalance, over-commitment and depressive episodes at work: evidence from the ELSA-Brasil Cohort Study. Int J Environ Res Public Health 2019, 16(17). 10.3390/ijerph16173025 PMC6747529 31438554

[24] Siegrist J (1996). Adverse health effects of high-effort/low-reward conditions. J Occup Health Psychol.

[25] Siegrist J, Li J: Work Stress and altered biomarkers: a synthesis of findings based on the effort-reward imbalance model. Int J Environ Res Public Health 2017; 14(11). 10.3390/ijerph14111373 PMC5708012 29125555

[26] Peter R, Siegrist J (1999). Chronic psychosocial stress at work and cardiovascular disease: the role of effort-reward imbalance. Int J Law Psychiatry.

[27] Siegrist J. Justice and Health. in *International encyclopedia of the social & behavioral sciences (second edition).* edn. Edited by Wright JD. Oxford: Elsevier; 2015: 928–931.

[28] Kunz C (2019). The influence of working conditions on health satisfaction, physical and mental health: testing the effort-reward imbalance (ERI) model and its moderation with over-commitment using a representative sample of German employees (GSOEP). BMC Public Health.

[29] Lau B (2018). Mental health among Norwegian priests: associations with effort-reward imbalance and overcommitment. Int Arch Occup Environ Health.

[30] Li J, Cheng Y, Siegrist J. Effort-reward imbalance at work and job dissatisfaction in Chinese healthcare workers: a validation study. Int Arch Occup Environ Health 2005; 78(3):198–204. 10.1007/s00420-004-0581-7 15838712

[31] Lai J, Ma S, Wang Y, Cai Z, Hu J, Wei N, Wu J, Du H, Chen T, Li R (2020). Factors associated with mental health outcomes among health care workers exposed to coronavirus disease 2019. JAMA Netw Open.

[32] Li L, Wan C, Ding R, Liu Y, Chen J, Wu Z, Liang C, He Z, Li C (2015). Mental distress among Liberian medical staff working at the China Ebola Treatment Unit: a cross sectional study. Health Qual Life Outcomes.

[33] Wang W, Bian Q, Zhao Y, Li X, Wang W, Du J, Zhang G, Zhou Q, Zhao M (2014). Reliability and validity of the Chinese version of the Patient Health Questionnaire (PHQ-9) in the general population. Gen Hosp Psychiatry.

[34] Zhang YL, Liang W, Chen ZM, Zhang HM, Zhang JH, Weng XQ, Yang SC, Zhang L, Shen LJ, Zhang YL (2013). Validity and reliability of Patient Health Questionnaire-9 and Patient Health Questionnaire-2 to screen for depression among college students in China. Asia Pac Psychiatry.

[35] Chin WY, Chan KT, Lam CL, Wong SY, Fong DY, Lo YY, Lam TP, Chiu BC (2014). Detection and management of depression in adult primary care patients in Hong Kong: a cross-sectional survey conducted by a primary care practice-based research network. BMC Fam Pract.

[36] Manea L, Gilbody S, McMillan D (2012). Optimal cut-off score for diagnosing depression with the Patient Health Questionnaire (PHQ-9): a meta-analysis. CMAJ.

[37] Spitzer RL, Kroenke K, Williams JBW, Löwe B (2006). A brief measure for assessing generalized anxiety disorder: the GAD-7. Arch Intern Med.

[38] He X, Li C, Qian J, Cui H, Wu W (2010). Reliability and validity of Chinese version of the Generalized Anxiety Disorder 7-item(GAD-7) scale in screening anxiety disorders in outpatients from traditional Chinese internal department. Chin Ment Health J.

[39] Löwe B, Decker O, Müller S, Brähler E, Schellberg D, Herzog W, Herzberg PY (2008). Validation and standardization of the Generalized Anxiety Disorder Screener (GAD-7) in the general population. Med Care.

[40] Rutter LA, Brown TA (2017). Psychometric properties of the Generalized Anxiety Disorder Scale-7 (GAD-7) in outpatients with anxiety and mood disorders. J Psychopathol Behav Assess.

[41] National Health Commission of the People’s Republic of China. China Health Statistics Yearbook, in *National Health Commission of the People’s Republic of China.* 2019.

[42] Plaisier I, de Bruijn JG, de Graaf R, ten Have M, Beekman AT, Penninx BW (2007). The contribution of working conditions and social support to the onset of depressive and anxiety disorders among male and female employees. Soc Sci Med.

[43] Rugulies R, Aust B, Madsen IE: Effort-reward imbalance at work and risk of depressive disorders. A systematic review and meta-analysis of prospective cohort studies. *Scand J Work Environ Health* 2017, 43(4):294–306. 10.5271/sjweh.3632 28306759

[44] Harris T (2001). Recent developments in understanding the psychosocial aspects of depression. Br Med Bull.

[45] Kendler KS, Hettema JM, Butera F, Gardner CO, Prescott CA (2003). Life event dimensions of loss, humiliation, entrapment, and danger in the prediction of onsets of major depression and generalized anxiety. Arch Gen Psychiatry.

[46] Seligman MEP: A series of books in psychology.Helplessness: On depression, development, and death. W H Freeman/Times Books/ Henry Holt & Co. W H Freeman/Times Books/ Henry Holt & Co.; 1975.

[47] Michael H (2018). Redekopp D E: The broader aims of career development: mental health, wellbeing and work. Br J Guid Couns.

[48] Xie H (2013). Strengths-based approach for mental health recovery. Iran J Psychiatry Behav Sci.

[49] Steger MF, Dik BJ, Duffy RD (2012). Measuring meaningful work: the work and meaning inventory (WAMI). J Career Assess.

[50] Van Vegchel N, de Jonge J, Bakker A, Schaufeli W (2002). Testing global and specific indicators of rewards in the effort-reward imbalance model: does it make any difference?. Eur J Work Organ Psy.

[51] China Daily: Novel coronavirus could soon build a home in a human being near us, in China Daily; 2020.

[52] Wang Z, Shi J, Gang X. Analysis on bottleneck and prospect of outstanding public health talents training in China (in Chinese). China Acad J Electron Publishing House 2020, 35(3).

[53] FRANCE24: France announces €1,500 bonus for health workers on Covid-19 front line. In FRANCE24*.*; 2020.

[54] Ministry of Finance of the People ’s Republic of China: Question and answer on financial support for covid-19 prevention and control policies and measures. In*.*The office of the ministry of finance; 2020.

[55] HHS Press Office: CDC to Award Over $560 Million to State & Local Jurisdictions in Support of COVID-19 Response. In*.*HHS Press Office; 2020.

[56] Li L, Ying W, Tiantian Z (2020). Thoughts on the construction of disease prevention and control system in the new era. Chin Health Resour..

