# Supplementary material for: Prevalence of mental health problems and associated factors among front-line public health workers during the COVID-19 pandemic in China: an effort–reward imbalance model-informed study
Source: BMC Psychol. 2021 Apr 12;9:55. doi: 10.1186/s40359-021-00563-0 (PMC8040352; doi:10.1186/s40359-021-00563-0)
Supplement: Supplementary file 1 — Additional file 1. The English version of online survey questionnaires, the confirmatory factor analysis and the exploratory factor analysis of ERI, and the supplementary analysis of results. [file 40359_2021_563_MOESM1_ESM.docx]

**Additional file 1. The English version of online survey questionnaires**

| The Survey for front-Line Public Health Workers During the COVID-19 Pandemic in China for Mental Health | | | | | | | | | | | | | | | | | | | | |
| --- | --- | --- | --- | --- | --- | --- | --- | --- | --- | --- | --- | --- | --- | --- | --- | --- | --- | --- | --- | --- |
| Dear Public Health Workers.  With the recent epidemic, we would like to express our sincere gratitude and respect for your efforts.  Through this survey, we hope to document the status of public health workers on the epidemic control front, the time they spend, the work they do, the stress they face, and more. If you are currently involved in the public health prevention and control work of this epidemic (such as prevention and control, health education, community public health work, etc., excluding clinical treatment, residential committee, civil police work), we invite you to participate in the survey, hoping that the results will make more people aware of the public health epidemic prevention and control work, and that the results will provide a basis for the improvement of the epidemic prevention and control system.  The survey is anonymous and voluntary and takes 8-10 minutes to participate. All information collected will be analyzed only in aggregate and in absolute confidentiality. If you agree to participate, please start filling out the questionnaire below. Again, thank you! | | | | | | | | | | | | | | | | | | | | |
| **I. General information** | | | | | | | | | | | | | | | | | | | | |
| **1.Your gender [Single-choice]** | | | | | | | ①Male | | | | | | | ②Female | | | | | | |
| **2.Your age [fill in the blank]** | | | | | | |  | | | | | | | | | | | | | |
| **3.How old is your youngest child? [Single-choice]** | | | | | | | | | | | | | | | | | | | | |
| ①No children | | | ②<1 year old | | | | ③1 to 2years old | | | | | | | ④3 to 5 years old | | | | | | |
| ⑤Elementary school students | | | | | | | ⑥Junior high school students | | | | | | | | | | | | | |
| ⑦High school students | | | | | | | ⑧University students and above | | | | | | | | | | | | | |
| **4.Type of unit you work for [fill in the blank]** | | | | | | | | | | | | | | |  | | | | | |
| **5.Your current profession (please fill in the most important)** | | | | | | | | | | | | | |  | | | | | | |
| **6.Your job title [Single-choice]** | | | | | | |  | | | | | | | | | | | | | |
| ① junior | | | ② intermediate | | | | ③ associate | | | | | | | ④ senior | | | | | | |
| ⑤ other (e.g., volunteer, unspecified) | | | | | | | | | | | | | | | | | | | | |
| **II. Epidemic prevention and control** | | | | | | | | | | | | | | | | | | | | |
| **7. What outbreak prevention and control field work are you currently involved in? [Multiple-choice]** | | | | | | | | | | | | | | | | | | | | |
| ①None | | | | | | | | | | | | | | | | | | | | |
| ②Patient transfer (face-to-face) | | | | | | ③Patient transfer (phone/video) | | | | | | | | | | | | | | |
| ④Interface transfer (face-to-face) | | | | | | ⑤Interface transfer (phone/video) | | | | | | | | | | | | | | |
| ⑥Interface tracking management | | | | | | ⑦Interface medical observation | | | | | | | | | | | | | | |
| ⑧Medical observation of infected area personnel | | | | | | | | | | | | | | | | | | | | |
| ⑨On-site extermination | | | | | | ⑩Sample collection | | | | | | | | | | | | | | |
| ⑪Sample transportation | | | | | | ⑫Health education | | | | | | | | | | | | | | |
| ⑬On-site guidance | | | | | | ⑭Logistic support | | | | | | | | | | | | | | |
| ⑮Hospitalization control | | | | | | ⑯Other (please add) | | | | | | | | | | | | | | |
| **8. What off-site work are you currently involved in outbreak prevention and control? [Multiple choice]** | | | | | | | | | | | | | | | | | | | | |
| ①None | | | | | | | | | | | | | | | | | | | | |
| ②Preparation of technical guidelines | | | | | | ③Online reporting/revision | | | | | | | | | | | | | | |
| ④Epidemic analysis | | | | | | ⑤Report writing | | | | | | | | | | | | | | |
| ⑥Laboratory testing | | | | | | ⑦Information and publicity | | | | | | | | | | | | | | |
| ⑧Other (please add) | | | | | | | | | | | | | | | | | | | | |
| **9. What organizations are you currently involved in coordinating outbreak prevention and control? [Multiple choice]** | | | | | | | | | | | | | | | | | | | | |
| ①None | | | | | | | | | | | | | | | | | | | | |
| ②Comprehensive coordination | | | | | | ③Information coordination | | | | | | | | | | | | | | |
| ④Quarantine point management and coordination | | | | | | | | | | | | | | | | | | | | |
| ⑤Technical training | | | | | | ⑥Supervision and inspection | | | | | | | | | | | | | | |
| ⑦Other (please add) _________________ | | | | | | | | | | | | | | | | | | | | |
| **10. If you are currently involved in outbreak prevention and control efforts not covered above, please add here [Single-choice]** | | | | | | | | | | | | | | | | | | | | |
| ①No additions | | | | | | ②Yes additions. _________________ | | | | | | | | | | | | | | |
| **11. Date your participation in outbreak prevention and control began** | | | | | | | | | | | | | | | | | |  | | |
| **12. Working days during the Spring Festival holiday (the 30^th^ to the 6^th^ day of the Lunar New Year) [Single-choice]** | | | | | | | | | | | | | | | | | | | | |
| ①0 | ②1 | ③2 | | ④3 | ⑤4 | | | | ⑥5 | | | | ⑦6 | | | | ⑧7 | | | |
| **13. Number of days of overnight work since joining the epidemic [Single-choice]** | | | | | | | | | | | | | | | | | | | | |
| ①0 | ②1 | ③2 | | ④3 | ⑤4 | | | | ⑥5 | | | | ⑦6 | | | | ⑧7 | | | |
| ⑨> 7 | | | | | | | | | | | | | | | | | | | | |
| **III. Anxiety and depression** | | | | | | | | | | | | | | | | | | | | |
| **14. How often have you been troubled by the following questions in the past two weeks?** | | | | | | | | | | | | | | | | | | | | |
|  | | | | | | | | Not at all | | | In less  than half the time | | | | More than half the time | | | | Almost every day | |
| 1) Feeling nervous, restless or irritable | | | | | | | | ① | | | ② | | | | ③ | | | | ④ | |
| 2) Inability to stop or control worries | | | | | | | | ① | | | ② | | | | ③ | | | | ④ | |
| 3)Worrying excessively about different things | | | | | | | | ① | | | ② | | | | ③ | | | | ④ | |
| 4) Difficult to relax | | | | | | | | ① | | | ② | | | | ③ | | | | ④ | |
| 5) Restlessness due to a lack of peace of mind | | | | | | | | ① | | | ② | | | | ③ | | | | ④ | |
| 6) Easily upset or irritable | | | | | | | | ① | | | ② | | | | ③ | | | | ④ | |
| 7)Feeling scared and thinking something terrible is going to happen | | | | | | | | ① | | | ② | | | | ③ | | | | ④ | |
| **15. How often have you been troubled by the following questions in the past two weeks?** | | | | | | | | | | | | | | | | | | | | |
|  | | | | | | | | Not at all | | | In less  than half the time | | | | More than half the time | | | | Almost every day | |
| 1) Lack of motivation or pleasure in doing things | | | | | | | | ① | | | ② | | | | ③ | | | | ④ | |
| 2) Feeling depressed, frustrated or hopeless | | | | | | | | ① | | | ② | | | | ③ | | | | ④ | |
| 3) Difficulty falling asleep, restless sleep, or excessive sleeping | | | | | | | | ① | | | ② | | | | ③ | | | | ④ | |
| 4) Feeling tired or lacking energy | | | | | | | | ① | | | ② | | | | ③ | | | | ④ | |
| 5) Loss of appetite or eating too much | | | | | | | | ① | | | ② | | | | ③ | | | | ④ | |
| 6) Feeling like shit, or a failure, or letting yourself or your family down | | | | | | | | ① | | | ② | | | | ③ | | | | ④ | |
| 7) Difficulty concentrating on things, such as when reading a newspaper or watching television | | | | | | | | ① | | | ② | | | | ③ | | | | ④ | |
| 8) moves or speaks so slowly that others  are already aware of it, or just the opposite more than usual fidgeting or fidgeting and moving around | | | | | | | | ① | | | ② | | | | ③ | | | | ④ | |
| 9) Having thoughts that are better than dying or hurting yourself in some way | | | | | | | | ① | | | ② | | | | ③ | | | | ④ | |
| **IV. Work Stress** | | | | | | | | | | | | | | | | | | | | |
| **16. ERI questionnaire** | | | | | | | strongly disagree | | | disagree | | neutral | | | | agree | | | | strongly agree |
| E-1 I have constant time-pressure due to a heavy workload. | | | | | | | ① | | | ② | | ③ | | | | ④ | | | | ⑤ |
| E-2 I sacrifice a lot for pandemic-related work. | | | | | | | ① | | | ② | | ③ | | | | ④ | | | | ⑤ |
| E-3 I have a lot of responsibility in pandemic -related work. | | | | | | | ① | | | ② | | ③ | | | | ④ | | | | ⑤ |
| E-4 I need to work overtime for pandemic-related work. | | | | | | | ① | | | ② | | ③ | | | | ④ | | | | ⑤ |
| R-1 Participating in pandemic-related work will improve my ability. | | | | | | | ① | | | ② | | ③ | | | | ④ | | | | ⑤ |
| R-2 Participating in pandemic-related work will help my future development. | | | | | | | ① | | | ② | | ③ | | | | ④ | | | | ⑤ |
| R-3 I receive the respect I deserve from my superiors. | | | | | | | ① | | | ② | | ③ | | | | ④ | | | | ⑤ |
| R-4 I receive the respect I deserve from my colleagues. | | | | | | | ① | | | ② | | ③ | | | | ④ | | | | ⑤ |
| R-5 I receive the respect I deserve from my service objects. | | | | | | | ① | | | ② | | ③ | | | | ④ | | | | ⑤ |
| R-6 I receive the respect I deserve from society. | | | | | | | ① | | | ② | | ③ | | | | ④ | | | | ⑤ |
| R-7 I participate in noble work. | | | | | | | ① | | | ② | | ③ | | | | ④ | | | | ⑤ |
| R-8 The work I am engaged in has important social significance. | | | | | | | ① | | | ② | | ③ | | | | ④ | | | | ⑤ |
| R-9 To be required to participate in pandemic-related work reflects your ability. | | | | | | | ① | | | ② | | ③ | | | | ④ | | | | ⑤ |
| OC-1 I am often faced with unsolvable difficulties in pandemic-related work. | | | | | | | ① | | | ② | | ③ | | | | ④ | | | | ⑤ |
| OC-2 As soon as I get up in the morning, I start thinking about work problems. | | | | | | | ① | | | ② | | ③ | | | | ④ | | | | ⑤ |
| OC-3 When I get home, I can easily relax and “switch off” pandemic-related work. | | | | | | | ① | | | ② | | ③ | | | | ④ | | | | ⑤ |
| OC-4 People close to me say I sacrifice too much for my job. | | | | | | | ① | | | ② | | ③ | | | | ④ | | | | ⑤ |
| OC-5 Pandemic-related work rarely lets me go; it is still on my mind when go to bed. | | | | | | | ① | | | ② | | ③ | | | | ④ | | | | ⑤ |

**Additional file 2. Exploratory factor analysis for the adapted effort-reward imbalance questionnaire**

| Subscale | Factor1 | Factor2 | Factor3 | Factor4 | Factor5 |
| --- | --- | --- | --- | --- | --- |
| Effort |  |  |  |  |  |
| E1. I have constant time-pressure due to a heavy workload. | **0.824** | 0.078 | 0.037 | -0.014 | 0.208 |
| E2. I sacrifice a lot for pandemic-related work. | **0.770** | -0.023 | 0.114 | 0.098 | 0.231 |
| E3. I have great responsibility in pandemic-related work. | **0.724** | -0.065 | 0.209 | 0.200 | 0.193 |
| E4. I need to work overtime for pandemic-related work. | **0.855** | 0.071 | 0.053 | -0.013 | 0.218 |
| Reward |  |  |  |  |  |
| Development |  |  |  |  |  |
| R1. Participating in pandemic-related work will improve my ability. | 0.056 | **0.739** | 0.106 | 0.421 | 0.049 |
| R2. Participating in pandemic-related work will help my future development. | -0.032 | **0.910** | 0.088 | 0.134 | 0.027 |
| Esteem |  |  |  |  |  |
| R3. I receive the respect I deserve from my superiors. | 0.198 | 0.118 | **0.782** | 0.096 | 0.012 |
| R4. I receive the respect I deserve from my colleagues. | 0.108 | 0.125 | **0.833** | 0.094 | -0.001 |
| R5. I receive the respect I deserve from my service objects. | 0.048 | 0.075 | **0.837** | 0.124 | 0.038 |
| R6. I receive the respect I deserve from society. | 0.014 | 0.057 | **0.838** | 0.145 | 0.048 |
| Job acceptance |  |  |  |  |  |
| R7. I participate in noble work. | 0.048 | 0.256 | 0.132 | **0.882** | 0.062 |
| R8. The work I am engaged in has important social significance. | 0.082 | 0.185 | 0.119 | **0.911** | 0.074 |
| R9. To be required to participate in pandemic-related work reflects your ability. | 0.019 | 0.525 | 0.149 | **0.634** | 0.080 |
| Over-commitment |  |  |  |  |  |
| OC1. I am often faced with unsolvable difficulties in pandemic-related work. | 0.233 | -0.011 | -0.050 | 0.002 | **0.686** |
| OC2. As soon as I get up in the morning, I start thinking about work problems. | 0.113 | 0.117 | 0.021 | 0.050 | **0.755** |
| OC3. When I get home, I can easily relax and “switch off” pandemic-related work. | 0.240 | 0.028 | 0.012 | 0.064 | **0.816** |
| OC4. People close to me say I sacrifice too much for my job. | 0.244 | 0.051 | 0.039 | 0.090 | **0.793** |
| OC5. Pandemic-related work rarely lets me go; it is still on my mind when go to bed. | 0.196 | 0.038 | 0.127 | 0.158 | **0.607** |
| Cronbach's Alpha | 0.852 | 0.749 | 0.863 | 0.874 | 0.824 |
| Initial Eigenvalues | 2.135 | 0.844 | 3.202 | 1.321 | 5.255 |
| Cumulative % of variance explained | 15.65 | 25.77 | 41.67 | 54.72 | 70.87 |

Note: Extraction Method: Principal Component Analysis. Rotation Method: Equamax with Kaiser Normalization.

**Additional file 3. The floor and ceiling effects of the adapted effort-reward imbalance questionnaire**

| Subscale | Item | Range | Mean | SD | Worst score  %(n) | Best score  %(n) |
| --- | --- | --- | --- | --- | --- | --- |
| Effort |  |  |  |  |  |  |
|  | E1 | 1-5 | 3.14 | 0.82 | 3.2(156) | 5.2 (251) |
|  | E2 | 1-5 | 3.31 | 0.83 | 1.4 (66) | 8.6(415) |
|  | E3 | 1-5 | 3.43 | 0.87 | 1.4 (69) | 11.8(574) |
|  | E4 | 1-5 | 3.10 | 0.84 | 3.3(162) | 5.1(249) |
| Reward |  |  |  |  |  |  |
| Development | R1 | 1-5 | 4.06 | 0.73 | 0.6(29) | 26.1(1267) |
|  | R2 | 1-5 | 3.60 | 0.97 | 2.4(115) | 17.9(866) |
| Esteem | R3 | 1-5 | 3.31 | 0.77 | 1.2(56) | 6.9(336) |
|  | R4 | 1-5 | 3.24 | 0.81 | 2.0(95) | 6.6(319) |
|  | R5 | 1-5 | 3.26 | 0.74 | 0.9(46) | 5.7(275) |
|  | R6 | 1-5 | 3.25 | 0.81 | 1.5(73) | 6.7(325) |
| Job acceptance | R7 | 1-5 | 4.30 | 0.70 | 0.4(17) | 42.0(2039) |
|  | R8 | 1-5 | 4.36 | 0.65 | 0.2(9) | 45.0(2181) |
|  | R9 | 1-5 | 4.06 | 0.79 | 0.5(22) | 30.4(1474) |
| Over-commitment |  |  |  |  |  |  |
|  | OC1 | 1-4 | 2.65 | 0.67 | 2.8(134) | 8.6(415) |
|  | OC2 | 1-4 | 2.80 | 0.70 | 2.9(142) | 13.3(647) |
|  | OC3 | 1-4 | 2.83 | 0.69 | 2.5(120) | 14.0(680) |
|  | OC4 | 1-4 | 2.89 | 0.62 | 1.7(82) | 12.9(626) |
|  | OC5 | 1-4 | 2.89 | 0.67 | 1.9(94) | 15.7(762) |

*Note*. The floor and ceiling effects of more than 70 % were considered to be *sig.*

**Additional file 4. Convergent validity of the confirmatory factor analysis for the adapted effort-reward imbalance questionnaire**

| Subscale | *AVE* | *CR* |
| --- | --- | --- |
| Effort | 0.596 | 0.854 |
| Development | 0.633 | 0.771 |
| Esteem | 0.614 | 0.864 |
| Job acceptance | 0.718 | 0.883 |
| Over-commitment | 0.500 | 0.829 |

*Note.* *AVE*: average variance extracted; *CR*: composite reliability. *AVE* values greater than 0.5 and *CR* values greater than 0.7, demonstrate an excellent convergent validity.

**Additional file 5. Discriminant validity of the confirmatory factor analysis for the adapted effort-reward imbalance questionnaire**

| Factor | Effort | Development | Esteem | Job acceptance | Over-commitment |
| --- | --- | --- | --- | --- | --- |
| Effort | 0.596 |  |  |  |  |
| Development | 0.053^***^ | 0.633 |  |  |  |
| Esteem | 0.249^***^ | 0.113^***^ | 0.614 |  |  |
| Job acceptance | 0.073^***^ | 0.287^***^ | 0.127^***^ | 0.718 |  |
| Over-commitment | 0.155^***^ | 0.168^***^ | 0.120^***^ | 0.055^***^ | 0.500 |
| Square root of *AVE* | 0.772 | 0.796 | 0.784 | 0.847 | 0.707 |

*Note.* *** *P* <0.001. Square root of *AVE* greater than the correlation coefficient value indicate that a good discriminant validity.

**Additional file 6. Logistic regression analysis of effort/over-commitment and reward (esteem) on depression (N = 4850)**

| Depression | Model 1 | | Model 2 | | Model 3 | |
| --- | --- | --- | --- | --- | --- | --- |
|  | *OR* | 95%*Cl* | *OR* | 95%*Cl* | *OR* | 95%*Cl* |
| **Socio-demographic characteristics** | | |  |  |  |  |
| Sex |  |  |  |  |  |  |
| Male | 1.00 | - | 1.00 | - | 1.00 | - |
| Female | 1.48 | (1.25,175)^***^ | 1.48 | (1.25,1.75)^***^ | 1.48 | (1.25,1.75)^***^ |
| Age | 0.96 | (0.95,0.97)^***^ | 0.96 | (0.95, 0.97)^***^ | 0.96 | (0.95, 0.97)^***^ |
| Having children under 6 years of age |  |  |  |  |  |  |
| No | 1.00 | - | 1.00 | - | 1.00 | - |
| Yes | 1.03 | (0.87, 1.23) | 1.13 | (0.94,1.37) | 1.03 | (0.86,1.23) |
| Job title |  |  |  |  |  |  |
| Junior | 1.00 | - | 1.00 | - | 1.00 | - |
| Intermediate | 1.13 | (0.93, 1.36) | 1.13 | (0.94, 1.37) | 1.13 | (0.94, 1.36) |
| Senior | 1.13 | (0.85, 1.50) | 1.12 | (0.85, 1.49) | 1.13 | (0.85, 1.50) |
| Others | 1.20 | (0.95, 1.52) | 1.20 | (0.95, 1.52) | 1.20 | (0.96, 1.52) |
| **Effort–Reward** | |  |  |  |  |  |
| Effort | 1.34 | (1.29,1.39)^***^ | 1.25 | (1.11, 1.41)^***^ | 1.34 | (1.29, 1.39)^***^ |
| Over-commitment | 1.16 | (1.12,1.20)^***^ | 1.16 | (1.12, 1.20)^***^ | 1.12 | (0.97, 1.30) |
| Esteem | 0.86 | (0.84,0.89)^***^ | 0.80 | (0.69, 0.91)^***^ | 0.83 | (0.71, 0.98)^*^ |
| **Interaction item** |  |  |  |  |  |  |
| Effort ×Esteem |  |  | 1.01 | (1.00,1.01) |  |  |
| Over-commitment × Esteem | | |  |  | 1.00 | (0.99, 1.01) |
| F-statistics |  | 748.24^***^ |  | 749.63^***^ |  | 748.42^***^ |
| Adjusted R2 |  | 14.89% |  | 14.91% |  | 14.89% |

Note. *** *P*<0.001.

**Additional file 7. Logistic regression analysis of effort/over-commitment and reward (development) on depression (N = 4850)**

| Depression | Model 1 | | Model 2 | | Model 3 | |
| --- | --- | --- | --- | --- | --- | --- |
|  | *OR* | 95%*Cl* | *OR* | 95%*Cl* | *OR* | 95%*Cl* |
| **Socio-demographic characteristics** | | |  |  |  |  |
| Sex |  |  |  |  |  |  |
| Male | 1.00 | - | 1.00 | - | 1.00 | - |
| Female | 1.49 | (1.26,1.76)^***^ | 1.49 | (1.26,1.76)^***^ | 1.48 | (1.25,1.75)^***^ |
| Age | 0.96 | (0.95,0.97)^***^ | 0.96 | (0.95, 0.97)^***^ | 0.96 | (0.95, 0.97)^***^ |
| Having children under 6 years of age |  |  |  |  |  |  |
| No | 1.00 | - | 1.00 | - | 1.00 | - |
| Yes | 1.03 | (0.86, 1.22) | 1.03 | (0.87,1.23) | 1.03 | (0.87,1.23) |
| Job title |  |  |  |  |  |  |
| Junior | 1.00 | - | 1.00 | - | 1.00 | - |
| Intermediate | 1.22 | (1.01, 1.47)^*^ | 1.16 | (1.02, 1.47)^*^ | 1.22 | (1.02, 1.47)^*^ |
| Senior | 1.20 | (0.91, 1.60) | 1.18 | (0.91, 1.60) | 1.20 | (0.90, 1.60) |
| Others | 1.18 | (0.94, 1.49) | 1.19 | (0.94, 1.49) | 1.19 | (0.94, 1.49) |
| **Effort–Reward** | |  |  |  |  |  |
| Effort | 1.29 | (1.24,1.33)^***^ | 1.33 | (1.27, 1.68)^***^ | 1.28 | (1.24, 1.33)^***^ |
| Over-commitment | 1.17 | (1.13,1.22)^***^ | 1.19 | (1.13, 1.22)^***^ | 1.37 | (1.17, 1.59)^***^ |
| Development | 0.87 | (0.83,0.92)^***^ | 0.91 | (0.86, 1.43) | 1.16 | (0.88, 1.53) |
| **Interaction item** |  |  |  |  |  |  |
| Effort ×Development |  |  | 0.98 | (0.97,1.00) |  |  |
| Over-commitment ×Development | | |  |  | 0.98 | (0.96, 1.00)^*^ |
| F-statistics |  | 686.38 ^***^ |  | 689.77 ^***^ |  | 690.61 ^***^ |
| Adjusted R2 |  | 13.66% |  | 13.72% |  | 13.74 |

Note. * *P* < 0.05; *** *P*<0.001.

**Additional file 8. Logistic regression analysis of effort/over-commitment and reward (job acceptance) on depression (N = 4850)**

| Depression | Model 1 | | Model 2 | | Model 3 | |
| --- | --- | --- | --- | --- | --- | --- |
|  | *OR* | 95%*Cl* | *OR* | 95%*Cl* | *OR* | 95%*Cl* |
| **Socio-demographic characteristics** | | |  |  |  |  |
| Sex |  |  |  |  |  |  |
| Male | 1.00 | - | 1.00 | - | 1.00 | - |
| Female | 1.48 | (1.25,1.75) ^***^ | 1.49 | (1.26,1.76)^***^ | 1.47 | (1.24,1.74)^***^ |
| Age | 0.96 | (0.95,0.97)^***^ | 0.96 | (0.95, 0.97)^***^ | 0.96 | (0.95, 0.97)^***^ |
| Having children under 6 years of age |  |  |  |  |  |  |
| No | 1.00 | - | 1.00 | - | 1.00 | - |
| Yes | 1.01 | (1.01, 1.46) | 1.01 | (0.85,1.20) | 1.01 | (0.85,1.21) |
| Job title |  |  |  |  |  |  |
| Junior | 1.00 | - | 1.00 | - | 1.00 | - |
| Intermediate | 1.21 | (1.01, 1.46)^*^ | 1.21 | (1.00, 1.46)^*^ | 1.21 | (1.00, 1.46)^*^ |
| Senior | 1.19 | (0.89, 1.58) | 1.19 | (0.90, 1.58) | 1.18 | (0.89, 1.57) |
| Others | 1.18 | (0.93, 1.49) | 1.18 | (0.94, 1.49) | 1.18 | (0.93, 1.48) |
| **Effort–Reward** | |  |  |  |  |  |
| Effort | 1.31 | (1.26,1.35)^***^ | 1.68 | (1.37, 2.06)^***^ | 1.31 | (1.26, 1.35)^***^ |
| Over-commitment | 1.19 | (1.15,1.24)^***^ | 1.20 | (1.15, 1.24)^***^ | 1.48 | (1.21, 1.81)^***^ |
| Job Acceptance | 0.82 | (0.79,0.86)^***^ | 1.08 | (0.87, 1.34) | 1.04 | (0.83, 1.30) |
| **Interaction item** |  |  |  |  |  |  |
| Effort ×Job Acceptance |  |  | 0.98 | (0.97,1.00)^*^ |  |  |
| Over-commitment ×Job Acceptance | | |  |  | 0.98 | (0.97,1.00)^*^ |
| F-statistics |  | 748.89^***^ |  | 755.27^***^ |  | 753.54^***^ |
| Adjusted R2 |  | 14.90% |  | 15.03% |  | 14.99 |

Note. * *P* < 0.05; *** *P*<0.001.

**Additional file 9. Logistic regression analysis of effort/over-commitment and reward (esteem) on anxiety (N = 4850)**

| Anxiety | Model 1 | | Model 2 | | Model 3 | |
| --- | --- | --- | --- | --- | --- | --- |
|  | *OR* | 95%*Cl* | *OR* | 95%*Cl* | *OR* | 95%*Cl* |
| **Socio-demographic characteristics** | | |  |  |  |  |
| Sex |  |  |  |  |  |  |
| Male | 1.00 | - | 1.00 | - | 1.00 | - |
| Female | 1.59 | (1.33,1.90)^***^ | 1.59 | (1.33,1.90)^***^ | 1.59 | (1.33,1.90)^***^ |
| Age | 0.98 | (0.97,0.99)^**^ | 0.98 | (0.97, 0.99)^**^ | 0.98 | (0.97, 0.99)^**^ |
| Having children under 6 years of age |  |  |  |  |  |  |
| No | 1.00 | - | 1.00 | - | 1.00 | - |
| Yes | 1.14 | (0.94, 1.38) | 1.14 | (0.94,1.38) | 1.14 | (0.94,1.38) |
| Job title |  |  |  |  |  |  |
| Junior | 1.00 | - | 1.00 | - | 1.00 | - |
| Intermediate | 0.87 | (0.71, 1.07) | 0.87 | (0.71, 1.06) | 0.87 | (0.71, 1.06) |
| Senior | 0.65 | (0.48, 0.89)^***^ | 0.65 | (0.48, 1.89)^**^ | 0.66 | (0.48, 0.90)^**^ |
| Others | 1.07 | (0.83, 1.37) | 1.07 | (0.83, 1.37) | 1.06 | (0.82, 1.36) |
| **Effort–Reward** | |  |  |  |  |  |
| Effort | 1.35 | (1.30,1.41) ^***^ | 1.41 | (1.22, 1.63) ^***^ | 1.35 | (1.30, 1.40) ^***^ |
| Over-commitment | 1.31 | (1.26,1.37) ^***^ | 1.31 | (1.26, 1.36) ^***^ | 1.51 | (1.27, 1.79) ^***^ |
| Esteem | 0.92 | (0.89,0.95) ^***^ | 0.96 | (0.81, 1.13) | 1.07 | (0.89, 1.30) |
| **Interaction item** |  |  |  |  |  |  |
| Effort ×Esteem |  |  | 1.00 | (0.99,1.01) |  |  |
| Over-commitment × Esteem | | |  |  | 0.99 | (0.98, 1.00) |
| F-statistics |  | 934.07 ^***^ |  | 934.35 ^***^ |  | 936.80 ^***^ |
| Adjusted R2 |  | 19.82% |  | 19.83% |  | 19.88% |

Note. ** *P*<0.01; *** *P*<0.001.

**Additional file 10. Logistic regression analysis of effort/over-commitment and reward (development) on anxiety (N = 4850)**

| Anxiety | Model 1 | | Model 2 | | Model 3 | |
| --- | --- | --- | --- | --- | --- | --- |
|  | *OR* | 95%*Cl* | *OR* | 95%*Cl* | *OR* | 95%*Cl* |
| **Socio-demographic characteristics** | | |  |  |  |  |
| Sex |  |  |  |  |  |  |
| Male | 1.00 | - | 1.00 | - | 1.00 | - |
| Female | 1.57 | (1.32,1.88)^***^ | 1.58 | (1.32,1.89)^***^ | 1.57 | (1.31,1.88) ^***^ |
| Age | 0.98 | (0.97,0.99)^***^ | 0.98 | (0.97, 0.99)^***^ | 0.98 | (0.97, 0.99) ^***^ |
| Having children under 6 years of age |  |  |  |  |  |  |
| No | 1.00 | - | 1.00 | - | 1.00 | - |
| Yes | 1.13 | (0.93, 1.37) | 1.14 | (0.94,1.38) | 1.14 | (0.94,1.38) |
| Job title |  |  |  |  |  |  |
| Junior | 1.00 | - | 1.00 | - | 1.00 | - |
| Intermediate | 0.92 | (0.75, 1.12) | 0.92 | (0.75, 1.12) | 0.92 | (0.75, 1.12) |
| Senior | 0.69 | (0.50, 0.94)^*^ | 0.69 | (0.50, 0.94)^*^ | 0.69 | (0.50, 0.94)^*^ |
| Others | 1.06 | (0.82, 1.36) | 1.05 | (0.82, 1.35) | 1.06 | (0.83, 1.36) |
| **Effort–Reward** | |  |  |  |  |  |
| Effort | 1.32 | (1.28,1.37)^***^ | 1.60 | (1.36, 1.88)^***^ | 1.32 | (1.28, 1.37)^***^ |
| Over-commitment | 1.34 | (1.28,1.39)^***^ | 1.34 | (1.29, 1.39)^***^ | 1.61 | (1.34, 1.93)^***^ |
| Development | 0.87 | (0.82,0.91)^***^ | 1.23 | (0.92, 1.66) | 1.23 | (0.87, 1.73) |
| **Interaction item** |  |  |  |  |  |  |
| Effort ×Development |  |  | 0.98 | (0.96,1.00)^*^ |  |  |
| Over-commitment ×Development | | |  |  | 0.98 | (0.96, 1.00)^*^ |
| F-statistics |  | 932.93 ^***^ |  | 938.75 ^***^ |  | 937.17 ^***^ |
| Adjusted R2 |  | 19.80% |  | 19.92% |  | 19.89% |

Note. * *P* < 0.05; *** *P*<0.001.

**Additional file 11. Logistic regression analysis of effort/over-commitment and reward (job acceptance) on anxiety (N = 4850)**

| Anxiety | Model 1 | | Model 2 | | Model 3 | |
| --- | --- | --- | --- | --- | --- | --- |
|  | *OR* | 95%*Cl* | *OR* | 95%*Cl* | *OR* | 95%*Cl* |
| **Socio-demographic characteristics** | | |  |  |  |  |
| Sex |  |  |  |  |  |  |
| Male | 1.00 | - | 1.00 | - | 1.00 | - |
| Female | 1.57 | (1.31,1.89)^***^ | 1.59 | (1.33,1.90)^***^ | 1.56 | (1.30,1.87)^***^ |
| Age | 0.98 | (0.97,0.99)^**^ | 0.98 | (0.97,0.99)^**^ | 0.98 | (0.97,0.99)^**^ |
| Having children under 6 years of age |  |  |  |  |  |  |
| No | 1.00 | - | 1.00 | - | 1.00 | - |
| Yes | 1.11 | (0.91, 1.34) | 1.11 | (0.92, 1.35) | 1.12 | (0.92, 1.35) |
| Job title |  |  |  |  |  |  |
| Junior | 1.00 | - | 1.00 | - | 1.00 | - |
| Intermediate | 0.91 | (0.74, 1.11) | 0.90 | (0.74, 1.10) | 0.90 | (0.74, 1.10) |
| Senior | 0.67 | (0.49, 0.92)^*^ | 0.68 | (0.49, 0.93)^*^ | 0.67 | (0.49, 0.92)^*^ |
| Others | 1.05 | (0.81, 1.35) | 1.05 | (0.81, 1.35) | 1.04 | (0.81, 1.34) |
| **Effort–Reward** | |  |  |  |  |  |
| Effort | 1.34 | (1.30,1.40)^***^ | 2.08 | (1.65,2.64)^***^ | 1.34 | (1.30,1.39)^***^ |
| Over-commitment | 1.36 | (1.31,1.42)^***^ | 1.37 | (1.31,1.43)^***^ | 1.87 | (1.44,2.43)^***^ |
| Job Acceptance | 0.82 | (0.79,0.86)^***^ | 1.32 | (1.03,1.70)^*^ | 1.17 | (0.88,1.57)^*^ |
| **Interaction item** |  |  |  |  |  |  |
| Effort ×Job Acceptance |  |  | 0.97 | (0.95,0.98)^***^ |  |  |
| Over-commitment ×Job Acceptance | | |  |  | 0.98 | (0.96, 1.00)^*^ |
| F-statistics |  | 978.06^***^ |  | 992.65^***^ |  | 984.45^***^ |
| Adjusted R2 |  | 20.76% |  | 21.07% |  | 20.89 |

Note. * *P* < 0.05; ** *P*<0.01; *** *P*<0.001.
